# Rehabilitation for Rhabdomyolysis Associated With Breast Cancer Treatment

**DOI:** 10.7759/cureus.8625

**Published:** 2020-06-15

**Authors:** Grace Burns, Christopher M Wilson

**Affiliations:** 1 Rehabilitation Services, Beaumont Health, Troy, USA; 2 Physical Therapy, Oakland University, Rochester, USA

**Keywords:** physical therapy, balance, fall, oncology, kidney, chemotherapy, weakness, pain, icu

## Abstract

Rhabdomyolysis can result in pain, physical limitations and may require hospitalization for medical management; however, little evidence has been reported on the efficacy of physical therapy (PT) interventions for this condition. Additionally, a review of literature on occurrences of rhabdomyolysis associated with cancer treatments yielded limited results. The purpose of this case report was to describe the acute and inpatient rehabilitation (IPR) examination, interventions, and outcomes for a patient with breast cancer and rhabdomyolysis. The patient was a 57-year-old female with a history of recurrent breast cancer who presented to the hospital with a chief complaint of bilateral thigh pain and weakness after the second cycle of chemotherapy and was diagnosed with rhabdomyolysis. After initially declining PT treatment during her first hospitalization, the patient was readmitted after a fall at home. She was hospitalized for 14 days with a transfer to IPR. The patient received a total of 12 days of PT (two acute care visits and 10 IPR visits) that included patient education, neuromuscular re-education, therapeutic exercise, and gait training. The patient was discharged home at a modified independent level with improvements in pain from 8/10 to 0/10 on the Numeric Pain Rating Scale. Functionally, she improved from ambulating 20 feet (6.1 m) to 600 feet (183 m); her Timed Up and Go score improved from 62 seconds to 10 seconds. Finally, her Berg Balance Scale improved from 31/56 to 55/56. Data from this case study suggest that PT management appears to improve functional outcomes for individuals diagnosed with rhabdomyolysis while undergoing chemotherapy, however, a holistic approach was necessary for progress to educate her on the importance of participation in PT with this condition. Further research is required to explore factors that influence rhabdomyolysis in the presence of chemotherapy as well as the recommended rehabilitation program for rhabdomyolysis.

## Introduction

Rhabdomyolysis is a potentially life-threatening disease characterized by three primary symptoms including myalgia, muscle weakness, and dark urine [1-2]. While the most widely recognized causes of rhabdomyolysis include overuse or injury, other documented causes include infections and chemotherapy drugs [3]. These mechanisms lead to muscle cell breakdown and the release of potentially toxic intracellular contents into the circulatory system which can lead to nephrotoxicity and acute kidney injury. Myoglobin specifically is released from damaged myocytes, causing occlusion and damage to the renal tubules [1, 4]. Renal failure can be measured by creatinine phosphokinase (CPK) levels increasing in blood serum following damage of the myocytes. Although there is not a commonly accepted diagnostic serum value of CPK for rhabdomyolysis, serum levels upon diagnosis “are usually at least five times the upper limit of normal, but range from approximately 1,500 to over 100,000 international units/L” [5].

A review of literature has not found a relationship between rhabdomyolysis in relation to docetaxel anhydrous (taxotere) and cyclophosphamide (which are the medications the patient in this case report received), however, it has been reported that rhabdomyolysis may be related to other drugs used during cancer treatment including imatinib, pemetrexed, erlotinib, and dexamethasone (e.g. steroids are often given in conjunction with docetaxel) [6-8]. An article by Huang et al. described a more severe case of life-threatening rhabdomyolysis in a 60-year-old patient with stage IV lung adenocarcinoma with spine metastasis [8]. Pemetrexed was given as a third line of chemotherapy with literature indicating this drug is generally well-tolerated. The authors hypothesized that the pemetrexed had induced rhabdomyolysis because three days after pemetrexed administration, the patient presented to the ED due to progressive weakness, generalized myalgia, inability to walk, respiratory distress, and a creatine phosphokinase (CPK) of 15,751 IU/L. The patient was then admitted to the ICU after emergent intubation. She remained intubated for one month and it was another month after extubation before she was able to functionally ambulate, however, it should be noted that the patient's disease-related morbidity may have impacted recovery. Huang et al. also described two additional cases of rhabdomyolysis during treatment for lung adenocarcinoma with erlotinib being the chemotherapy drug used in both cases [8]. Similar symptoms were reported, however, respiratory distress did not develop in these two cases.

Docetaxel and cyclophosphamide are often delivered as combination therapy. Docetaxel anhydrous belongs to the class of chemotherapy drugs called plant alkaloids, which are cell-cycle specific [9]. This antimicrotubule agent disrupts the cell’s apparatus for dividing and eventually leads to cell death [9]. Anemia and leukopenia are common findings after docetaxel. Nausea, fluid retention, and peripheral neuropathy are also relatively common; however, steroid administration has been shown to mitigate fluid retention [10]. Docetaxel is also associated with fatigue and weakness as well as less common side effect of myalgia and arthralgia [9]. Cyclophosphamide is an alkylating agent that has been cited to cause arthralgias, dyspnea, pericardial effusions, and renal failure [11]. 

Although rehabilitation for patients with rhabdomyolysis is not widely researched, Schleich et al. proposed a therapeutic regimen with four phases for athletes with exertional rhabdomyolysis [12]. The first phase was returning to normal activities for two weeks with close monitoring. The second phase had the individual initiating dynamic warmups, aquatic jogging, and stretching. When the individual could tolerate this without soreness or pain, phase 3 included light resistance activities, stationary bicycling, and body weight resistance activities to weak muscle groups. Phase 4 allowed the individual to return to the regular exercise routine with initiation of resistance training at 20%-25% of one-repetition maximum, agility exercises, and running [12]. No articles could be located related to rehabilitation after chemotherapy-induced rhabdomyolysis. A key component of returning to physical activity after musculoskeletal pain is the concept of mitigating fear-avoidance behavior and pain-avoidance behavior, which may result in a patient being reluctant or fearful to participate in rehabilitation [13]. One mechanism available to clinicians is the use of cognitive behavioral therapy (CBT) to provide education on the role of psychological, physiological, environmental, or contextual factors on pain and fear and how these factors can be mitigated through a variety of physical and behavioral modifications [14].

The purpose of this case report was to describe the acute and inpatient rehabilitation (IPR) examination, interventions, and outcomes for a patient with breast cancer and rhabdomyolysis. The patient provided written informed consent for dissemination of this case report.

## Case presentation

Patient history

The patient was a 57-year-old female with recurrent breast cancer who presented to a community hospital with a chief complaint of bilateral thigh pain and weakness. She was initially diagnosed with left breast cancer at age 50. The cancer was determined to be estrogen receptor (ER) positive stage I, grade 1 breast cancer, and the patient underwent adjuvant chemotherapy [which included doxorubicin (adriamycin)] and mastectomy followed by tamoxifen for five years. This was then followed by letrozole for approximately two years. Seven years from her initial diagnosis, she developed left axillary lymphadenopathy and was given the diagnosis of biopsy-proven recurrent metastatic breast cancer ER positive 90%, progesterone receptor (PR) negative, HER2/neu negative, and she underwent a left axillary lymphadenectomy. Doxorubicin was recommended as an initial treatment course, however, the patient adamantly refused due to her concern for cardiotoxicity, even when it was offered at a lower dose. She was instead started on chemotherapy with docetaxel (taxol) and cyclophosphamide (cytoxan) every 21 days. The second cycle was given with pegfilgrastim (neulasta) to stimulate growth of white blood cells in the body and has the common side effect of ostealgia [15].

Shortly after the second cycle of chemotherapy, the patient presented to the emergency center reporting significantly worse pain as compared to the first round of chemotherapy. She stated the pain began three days after chemotherapy treatment, and pain medications did not provide relief. Increased pain was noted with ambulation and palpation of thigh muscles. Her CPK was 3,607 units/L at the time of original admission for rhabdomyolysis.

Clinical presentation

The patient was referred to physical therapy (PT) after she was given a diagnosis of rhabdomyolysis, three days after admission. Upon the physical therapist’s initial encounter, the patient deferred a physical examination due to fatigue and pain. A PT evaluation was performed on the fourth day of admission to the hospital’s oncology unit.

Upon examination, the patient was found to have generalized weakness in left triceps, wrist and finger extension, finger flexion, and dorsal interossei; it was hypothesized that this weakness may have been due to postsurgical changes from the lymph node dissection approximately four weeks ago. Right hand weakness was noted to a lesser extent. Her bilateral quadriceps strength was rated at 3/5 with pain upon manual muscle testing, however, she was independent in functional mobility including bed mobility, transfers, and gait. A MRI assessment of the brachial plexus bilaterally was ordered to assess for a cause of the new onset of weakness (e.g. metastasis or edema). This MRI never occurred as the patient could not tolerate it secondary to anxiety.

In the initial PT evaluation, education was provided on use of assistive device and gradual strengthening. The patient was resistant to use of walker and declined stair training to get into her home. After the initial session, the patient demonstrated apprehension and declined any further PT sessions in the hospital. She reported that she was walking the nursing unit independently throughout the day, and would no longer need PT. Subsequently, she was discharged from PT services per her request. After 15 days in the hospital and only one PT session, she was discharged home. Upon attempting entry into her home, while approaching the steps, she sustained a fall. After this fall, she was readmitted to the hospital that same day.

During this second hospitalization, a referral to PT was initiated in the emergency center. During the second PT examination in the emergency center’s medical observation unit, the patient was found to have hyperalgesia preventing the physical therapist from performing light touching to the patient’s legs without extreme pain. Therefore, manual muscle testing was not completed. Functional assessment showed that the patient required minimal assistance for sit-to-stand transfers with use of her upper extremities. While the Activity Measure for Post-Acute Care (AM-PAC) six clicks showed patient would be able to return home [16], her five times sit-to-stand test of 32 seconds indicated further rehabilitation was needed as the mean for her age group is 11.4 seconds [17]. Additionally, the Timed Up and Go (TUG), a quick measure of balance and mobility, was 62 seconds; this was compared to available normative TUG values for females nearest her age (60-69 years old) was eight seconds. [18]. The Berg Balance score of 31/56 placed her in a category consistent with a moderate risk for falls [19]. A consult to physical medicine and rehabilitation for possible IPR was placed.

**Figure 1:**
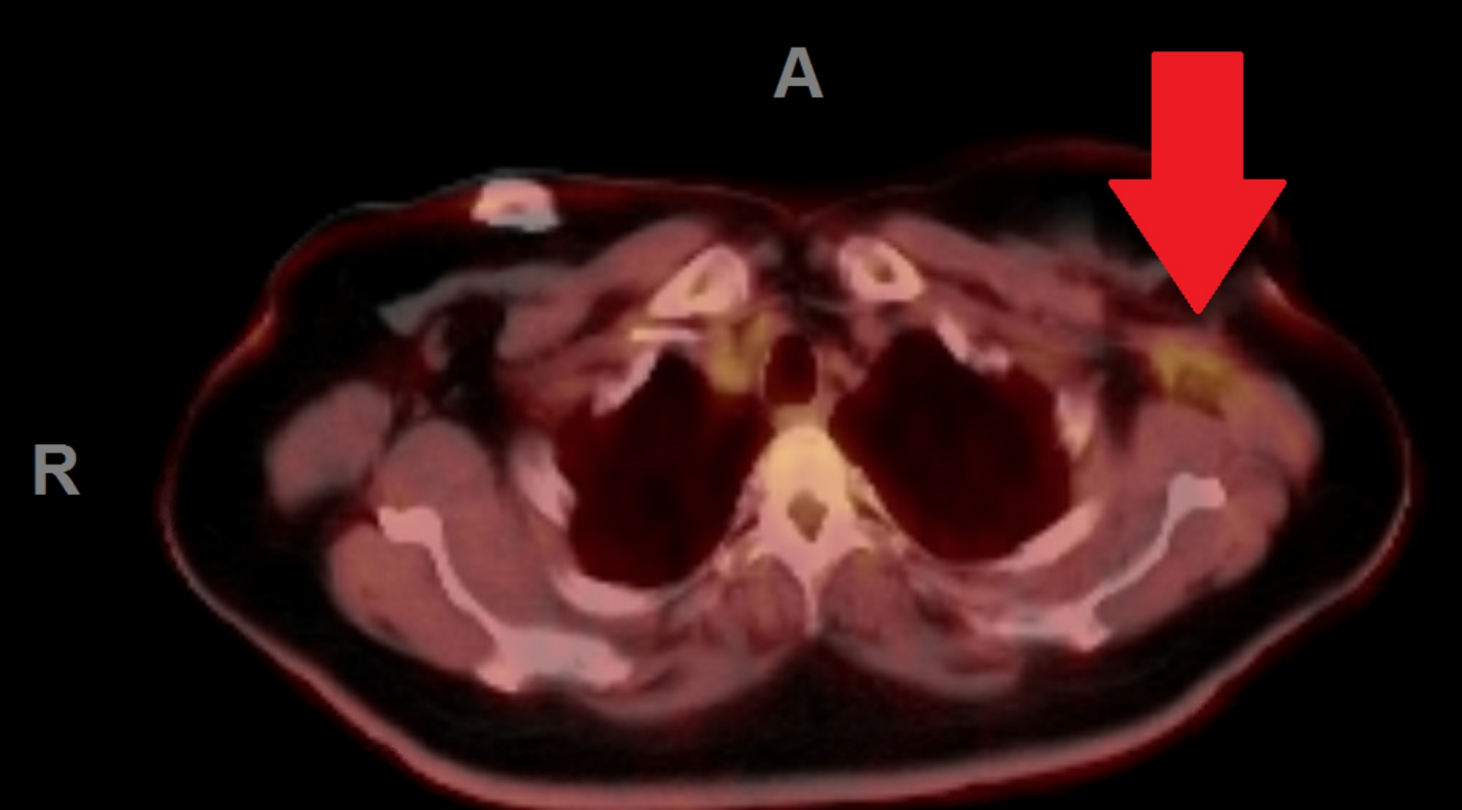
Postsurgical inflammation to the left axilla on PET scan. Red arrow indicates location of inflammation. A = anterior; R = right PET, positron emission tomography

**Figure 2:**
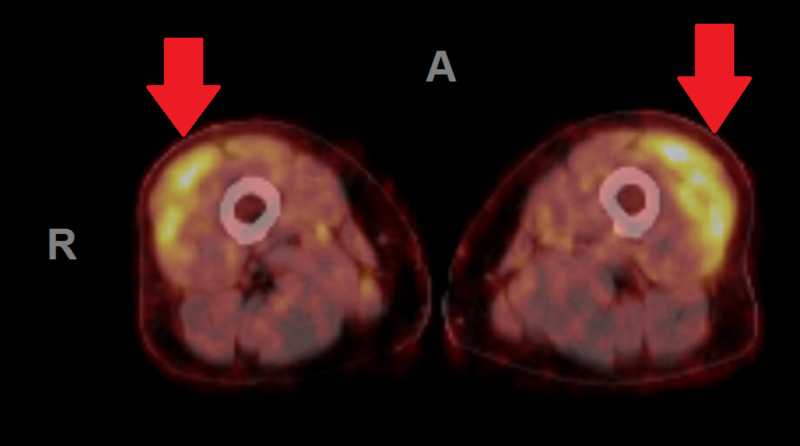
Bilateral anterior thigh inflammation and intramuscular inflammation on PET scan. Red arrows indicate areas of inflammation. A = anterior; R = right PET, positron emission tomography

During the second hospitalization, the patient underwent a positron emission tomography (PET) scan to assess for the source of hyperalgesia and assess for metastatic disease. An area of increase inflammation and post-surgical tissue response was found in the left axilla which may have been contributing to her left arm weakness (Figure 1). In addition, the PET scan demonstrated increased metabolic activity in the anterior thighs (Figure 2).

 

Interventions

Acute Care Hospitalization

Given the patient’s hyperalgesia, the first PT session consisted of deep breathing and calming procedures as well as CBT interventions including cognitive reframing of her pain as well as education on the neuroscience of pain. She was instructed to perform deep breathing throughout the day, as well as providing tactile stimulation to her bilateral thighs. Her home exercise program included supine heel slides and diaphragmatic breathing.

The next (and last acute care) PT session consisted of a seated exercise stepper (NuStep LLC, Ann Arbor, Michigan, USA) at workload level of 1-2 for 10 minutes and low repetitions (5-10) of closed- and open-chain lower extremity exercises consisting of heel/toe raises, mini-squats, hip abduction, and hamstring curls. Consistent education was provided on the purpose of exercise with positive framing to help reduce anxiety. The patient was agreeable to gait training with an assistive device after an explanation was provided that increased confidence and efficacy with using a walker would facilitate reducing the need for family assistance during gait upon return home.

After two sessions of PT in the acute care setting, the patient was then transferred to the hospital’s IPR unit. The progression of interventions was divided into four phases with the interventions initiated in IPR denoted by an asterisk (Table 1). Each of the phases lasted approximately two visits in IPR and the patient was transitioned to the next phase when she was generally asymptomatic with the activities performed in the current phase. 

**Table 1:** Summary of interventions in acute care and IPR. *Interventions initiated in IPR IPR, inpatient rehabilitation

	Phase 1	Phase 2	Phase 3	Phase 4
Therapeutic exercise	Open chain seated exercise, basic home exercise program	Closed chain exercise, eccentric control, ambulation	Light resistant theraband exercise*, Nustep low load 1-2	Increased resistance with closed chain standing exercise*, Nustep level 3*
Neuromuscular Re-education	Tactile stimulation of lower extremities, cognitive reframing for anxiety management	Standing static balance	Standing dynamic balance*	Varying surfaces including foam cushion*, Wii balance*, 2.2 lb (1 kg) medicine ball*
Gait training	Proper use of rolling walker	Step training with walker, dynamic gait training*	Gait training with cane*	Initiate gait training without assistive device*
Patient education	Relaxation strategies, neuroscience of pain, deep breathing	Safety in transfers and need for walker with ambulation	Education on rating of perceived exertion and cancer related fatigue*	Home recommendations and home exercise program*

Inpatient Rehabilitation

In the IPR setting, PT focused on progression of therapeutic exercise to light resistance exercises with elastic exercise bands or 2 lbs (0.91 kg) ankle weights. Closed chain strengthening exercises were introduced and included standing marching, hip abduction, partial squats, hamstring curls as well as step-taps on to a six-inch step. Two sets of 10 repetitions of each exercise was performed bilaterally but ceased before complete fatigue to avoid additional muscular. Aerobic exercise was administered via recumbent bike, which was incrementally increased to maximum of 16 minutes with the resistance set to “moderate” per the patient’s subjective report.

Neuromuscular re-education included standing static and dynamic balance activities on a blue foam balance pad with and without upper extremity support. This was initially performed with eyes opened then with eyes closed, then finally with eyes closed with a narrow base of support (i.e. feet together). Ambulation on a balance beam was then added with upper extremity support for approximately 15 feet (4.5 m) for three to four repetitions. Finally, the patient performed dynamic balance and righting activities while standing on a Nintendo Wii (Nintendo Co, Ltd. Kyoto, Japan) balance pad without UE support, this was advanced to include multiplanar weight shifting outside of the base of support.

Gait training in IPR was progressively challenged via dynamic gait activities including turning her head or body, speed variation, and ambulation over or around obstacles while holding a rolling walker. After she was able to accomplish these tasks, she was progressed to gait training with cane and without an assistive device. Gait distance was substantially shorter with a cane or no assistive device as she demonstrated increased lateral sway and difficulty clearing the left lower extremity during swing phase noted at approximately 75 feet (23 m) of ambulation. Education not only was provided to the patient, but to the patient’s family on stair management, home recommendations for durable medical equipment, and a home exercise program.

Outcomes and follow-up

The patient was discharged home at a modified independent level 29 days after her initial presentation to the hospital and 13 days after readmission to the hospital. Her total inpatient rehab stay was 11 days with marked improvement in functional status as well as subjective reports in confidence and pain. The patient demonstrated improvement with pain at 0/10 upon discharge home and her quadriceps and hip strength improved bilaterally; however, it was unclear as to why her left leg remained comparatively weaker than the right leg upon discharge (Table 2). Her TUG test improved to 10 seconds. She was able to ambulate community distances with a walker at discharge. The Berg Balance Scale results improved to indicate a low fall risk at 55/56.

**Table 2:** Summary of patient outcomes. MMT = manual muscle test, R = right, L = left, TUG = Timed Up and Go

	Pain	Quadriceps MMT R/L	Hip flexor MMT R/L	TUG score (seconds)	Distance ambulated (feet)	Berg Balance Scale
Initial	8	4-/3+	3+/3-	62	20	31/56
Discharge	0	4/4-	4/3	10	600	55/56

## Discussion

The purpose of this case study was to describe the acute and IPR examination, interventions, and outcomes for a patient with breast cancer and rhabdomyolysis. A prompt diagnosis of rhabdomyolysis will facilitate proper treatment and rehabilitation. Considerations for differential diagnosis should be taken into account as the patient did receive pegfilgrastim which can carry the side effect of bone pain and both docetaxel and cyclophosphamide are cited to cause arthralgias [9, 11, 15]. As many rehabilitation professionals may be practicing in an ambulatory or outpatient clinic, lab values such as CPK may not be readily available. In these cases, close communication of any unexpected weakness, pain, or functional decline should be relayed to the patients’ medical oncologist promptly for further diagnostic workup and treatment.

Not only was this patient experiencing physical impairments, it was felt that her anxiety and fear were substantial barriers when the initial PT referral was initiated. The physical therapist had to provide comprehensive education and coaching to facilitate performance in her rehabilitative regimen. Without cognitive reframing, the efficacy of a traditional rehabilitation program was questionable. It has been suggested in the literature that inclusion of CBT in a traditional rehabilitation program may improve patient compliance with exercise and participation in general [14]. Nielsen et al. described the two major goals of CBT in rehabilitation: 1. “to enhance patients' understanding of how psychological, social, and environmental factors influence their daily pain, function, and quality of life.” and 2. providing “systematic training to increase the patient's ability to effectively cope with pain through learning, practicing, and applying a range of behavioral skills (e.g., pleasant activity scheduling, activity pacing to achieve graded activation, problem solving, relaxation training) and cognitive skills (e.g., cognitive restructuring, pleasant imagery, cognitive distraction techniques such as focal point and counting)” [14]. Further study of utilization of CBT in oncology rehabilitation would be beneficial. Perhaps with inclusion of CBT in the therapy sessions during initial hospital admission, the patient would have been willing to participate and even avoid a fall and readmission. Anxiety related to recurrence of disease and complications experienced related to chemotherapy is likely not unique to this patient. As her chemotherapy had precipitated rhabdomyolysis and she had already declined doxorubicin, her anxiety was anticipated to be a substantial determinant of future success in cancer treatments and rehabilitation. Management of anxiety will be a critical component of management of her clinical care. Finally, cancer-related fatigue (a common symptom in cancer survivors) had likely further complicated and compounded the muscle weakness from rhabdomyolysis [20]. These interacting factors may have had a substantial impact on this patient’s quality of life and reinforces the importance of establishment of a comprehensive and ongoing cancer survivorship plan [20].

Although within the literature, there have not been conclusive studies to establish the relationship between medications used during cancer care and rhabdomyolysis, there are case studies in the literature linking pharmacological causes of this pathology [2, 6-8]. Given the various causes of rhabdomyolysis, continued documentation of these occurrences is warranted to assess for trends. Given the physiological effects of cancer treatments including chemotherapy and radiation, research involving unusual complications of these treatments should continue to be presented and published.

## Conclusions

Physical rehabilitation has an increasingly critical role in cancer treatment and rehabilitative regimens are useful to restore normal physical function from the cancers or side effects of its treatment. Physical activity and rehabilitation treatments are beneficial in safely delivering strengthening and aerobic exercise to minimize immobility and ensure safety. Exercise is also important in improving cancer-related fatigue, improving quality of life, and assisting in mitigating unnecessary delays in cancer treatment. Additionally, a patient experiencing significant physical and emotional stress is best approached holistically with a rehabilitation program with CBT inclusive of education on pain itself, safety, and exercise while empowering the patient and minimizing stress. After just over a month in the hospital, the patient was able to achieve discharge home at a safe level and prepare for her ongoing cancer treatment. A holistic, longitudinal approach (including CBT) and coordination of transitions from acute care to IPR provided the circumstances for effective physical rehabilitation in helping this patient improve her independence, safety, and confidence.
